# Design and Optimization Lattice Endoprosthesis for Long Bones: Manufacturing and Clinical Experiment

**DOI:** 10.3390/ma13051185

**Published:** 2020-03-06

**Authors:** Pavel Bolshakov, Ivan Raginov, Vladislav Egorov, Regina Kashapova, Ramil Kashapov, Tatyana Baltina, Oskar Sachenkov

**Affiliations:** 1 Institute of Mathematics and Mechanics, Kazan Federal University, 420008 Kazan, Russia; bolshakov-pavel@inbox.ru (P.B.); tvbaltina@gmail.com (T.B.); 2 Institute of Engineering, Kazan Federal University, 420008 Kazan, Russia; raginovi@mail.ru (I.R.); reginasharapova13@mail.ru (R.K.); kashramil.88@mail.ru (R.K.); 3 Federal Center for Toxicological, Radiation and Biological Safety, 420075 Kazan, Russia; vladislav.e@inbox.ru; 4 Kazan National Research Technical University named after A.N. Tupolev, 420111 Kazan, Russia

**Keywords:** lattice endoprosthesis, long bone, additive manufacturing

## Abstract

The article is devoted to the construction of lattice endoprosthesis for a long bone. Clinically, the main idea is to design a construction with the ability to improve bone growth. The article presents the algorithm for such a design. The construction should be produced by additive manufacturing. Such an approach allows using not only metallic materials but also ceramics and polymers. The algorithm is based on the influence function as a method to describe the elementary cell geometry. The elementary cell can be described by a number of parameters. The influence function maps the parameters to local stress in construction. Changing the parameters influences the stress distribution in the endoprosthesis. In the paper, a bipyramid was used as an elementary cell. Numerical studies were performed using the finite element method. As a result, manufacturing construction is described. Some problems for different orientations of growth are given. The clinical test was done and histological results were presented.

## 1. Introduction

Currently, arthroplasty is widespread in surgery. The main problem of arthroplasty is considered that the implant remains in the body after the operation. This fact leads to bone growth problems in the area of an endoprosthesis. The best solution can be to produce an endoprosthesis using biomaterials. Additionally, using additive manufacturing can increase the quality of arthroplasty. In this case, one of the possible solutions can be lattice design. Such constructions, on one hand, can have sufficient strength, on another hand, stimulate bone growth (e.g., by putting bone material inside). Nowadays, there are technical problems to use biomaterials in manufacturing. However, the first step is to develop the design principals of such constructions. On the one hand, the construction should be strong enough, on the other hand, the construction should provide bone growth.

To improve the technical state lattice structures are investigated until nowadays. The dependence between different geometry of the lattice, mechanical properties, and biological adaptive is being researched [1,2,3]. Additive manufacturing allows the production of irregular lattice constructions. The main idea of such construction is to mimic bone geometry and stiffness properties. Additionally, the porous structure can increase the quality of local bone growth. Traditionally manufactured lattice constructions using casting and forging techniques cannot achieve the levels of additive manufacturing. However, problems appear because of melting: A manifestation of brittle properties and geometry deviations [4,5,6,7]. Different materials and melting modes are investigated to improve the quality of constructions [8,9,10]. Nowadays there are papers with a wide review focuses on three-dimensional-printed technics and related composite systems were performed [11]. In the composite system, the calcium phosphate phase is combined with other ceramics or polymers improving the mechanical properties and/or imparting special extra-functionalities. Nowadays, additive technologies are actively used in the manufacture of individual implants. The most widely used technology is selective laser melting. This technology allows the manufacture of high-precision implants with a developed rough surface. Moreover, one of the important advantages of this technology compared to traditional ones is the ability to create mesh structures with specified cell parameters. Therefore, studies are being carried out all over the world to obtain various network structures using selective laser melting methods and their bio and mechanical properties. However, interest does not subside because of good usage potential in biomedicine [12,13,14,15]. Patient-based medicine became more widespread with the growth of technical abilities in computational mechanics and computed tomography scan quality [16,17,18]. Nowadays, it is possible to not only perform simulation of surgical treatment [19,20,21] but design the endoprosthesis [14,16] and predict biomechanical reaction [22,23,24]. Joint usage of the patient CT-data, patient-based design of the endoprosthesis, and additive manufacturing can increase the quality of the orthopedic treatment. The goal of the work is the construction of a lattice endoprosthesis for long bones.

## 2. Materials and Methods

**Figure 1:**
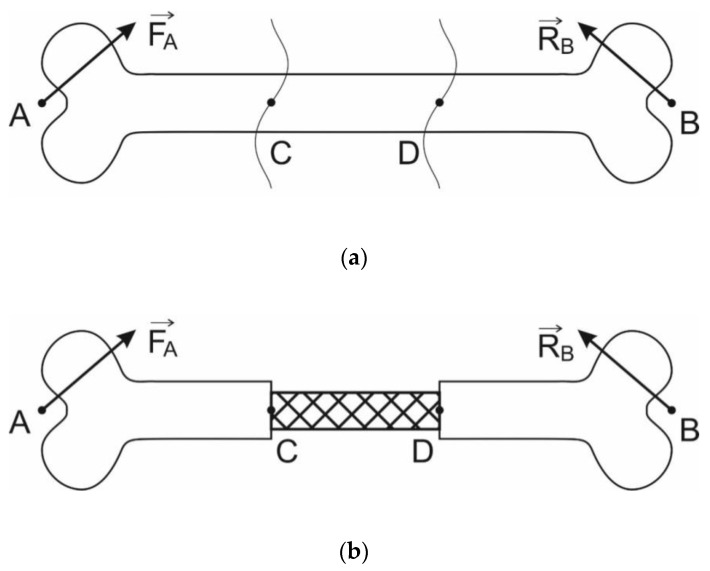
Scheme of long bone before (**a**) and after mounting lattice implant (**b**). Force F applied at point A, force R applied at point B. Section CD should be replaced by endoprosthesis.

**Figure 2:**
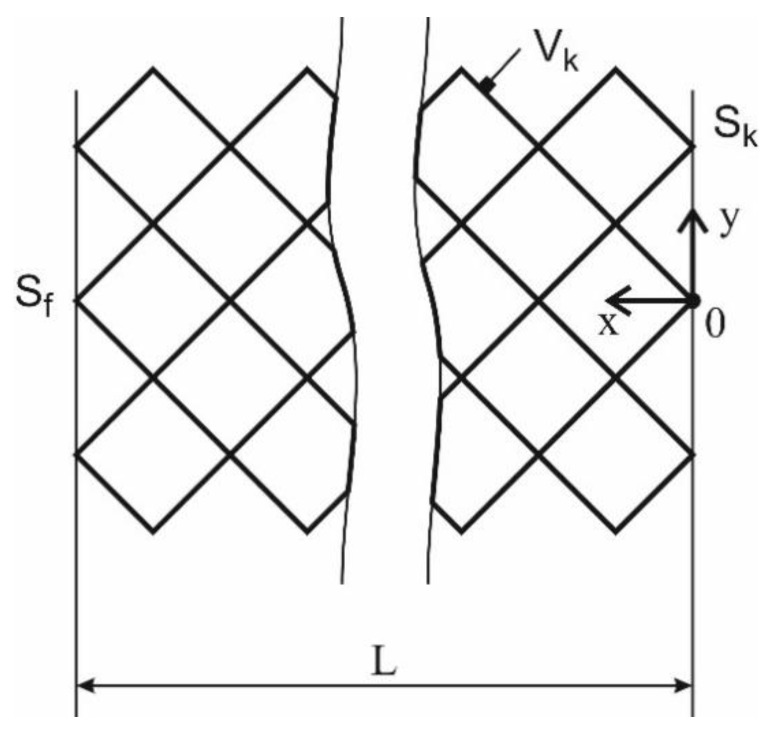
The design of lattice endoprosthesis.

The form of the endoprosthesis should allow the insertion of bone material into the endoprosthesis to improve the growth of bone tissue [6]. In Figure 1, the long bone (a) and long bone with lattice endoprosthesis (b) are schematically shown. According to weight and physical activity (walk, run, etc.), different forces act on the bone. Hexagonal bipyramid was chosen for an elementary cell of the lattice endoprosthesis [7,10]. Such geometry allows containing a sufficient amount of bone material, which potentially can improve bone tissue growth [13,22,23]. During the usage of the implant, bending and compression is experienced. The implant geometry was defined as follows: The endoprosthesis consists of sets of blocks that are linked in the longitudinal direction of the endoprosthesis (see Figure 2). Each block consists of a set of elementary lattice cells. The geometry of the hexagonal bipyramid allows arranging the cells in blocks most compactly. Such a design has good properties for additive manufacturing [2,7].

The general problem of the stress-strain state (SSS) of the endoprosthesis can be formulated as follows:

(1) $$\nabla \tilde{\sigma }=0\;\forall x\in V$$

(2) $$\tilde{\varepsilon }=\frac{1}{2}\left(\nabla \vec{u}+\nabla {\vec{u}}^{T}\right)\;\forall x\in {\bar{V}}_{k}$$

(3) $$\tilde{\sigma }=\tilde{\tilde{E}}:\tilde{\varepsilon }\;\forall x\in {\bar{V}}_{k}$$

(4) $$\vec{u}=0\;\forall x\in S_{k}$$

(5) $$\tilde{\sigma }\cdot \vec{n}=\vec{F}\;\forall x\in S_{f}$$

where *V_k_*—the investigated volume, *σ*—stress tensor, *ε*—strain tensor, *E*—stiffness tensor, *S_k_*—area with kinematic boundary conditions, *S_f_*—area with static boundary conditions, *u*—displacement vector, *n*—normal to the area with static boundary conditions.

Obviously, the loading of all elementary cells is uneven. Varying the geometry of each elementary cell, it is possible to change the stress state in the implant. For this purpose, it is necessary to determine the relationship between the dimensions of an elementary cell and its stress state. Let us call such a connection the influence function. For the purpose of designing an endoprosthesis, the following algorithm can be formulated:

(1) Determine the initial length of all blocks.
(2) Apply workloads and boundary conditions to the structure.
(3) Solve the problem of SSS.
(4) Estimate the highest stresses in each block.
(5) Change the length of the blocks according to the influence functions and design constraints.
(6) Check the stop condition for the iterations, otherwise, go to step 3.

Various factors can be understood as the design constraints in the algorithm. For example, the total length of the implant or minimal size of the elementary cell can be given. For finite element analysis, the Ansys v. 14 software (ANSYS, Inc., Canonsburg, PA, USA) was used.

**Figure 3:**
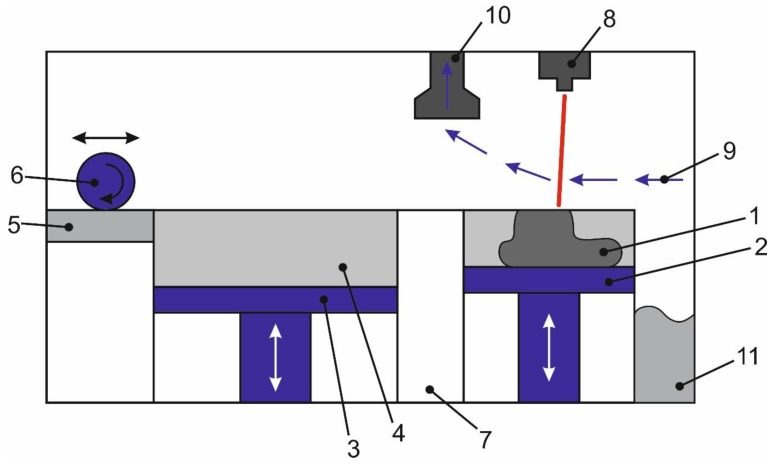
The scheme of the working chamber ProX 300: 1—Product, 2—product growing platform, 3—metal powder feeding platform, 4—metal powder, 5—area for cleaning the roller from powder, 6—roller for applying powder, 7—a partition between the construction zone and the powder storage hopper, 8—optical laser beam control system, 9—blowing gas, 10—output of combustion products, 11—storage bin for excess powder.

The experiments used a selective laser melting system known as ProX300 3D Systems (PROX 300, 3D Systems, Rock Hill, SC, USA). Figure 3 shows the diagram of the inner chamber, the length of which is 1500 mm, the height of 800 mm. The chamber is hermetic and allows creating an atmosphere of inert gas (9), in the chamber to the left is a metal roller for applying powder (6), it has the possibility of horizontal movement throughout the chamber. In the middle, there is a box (3) with a powder inside which a movable piston is located, on the right (2) is a construction zone consisting of a movable piston with a platform and a laser (8) mounted above it. The cycle for the construction of one layer consists of the following steps: Lifting the central piston by 60 μm, lowering the working platform of the construction by 40 μm, moving the roller through the central box, moving the excess powder to the working platform of construction, and scanning the surface of the powder with the laser. During the scan, combustion products can be generated, which are carried away by the flow of gas into the dust container.

The ProX 300 is equipped with a fiber Nd: YAG laser power of 500 W, with a wavelength of 1080 nm, the laser spot area was 20 μm. The size of the construction zone is x—250 mm, y—250 mm, z—300 mm, the minimum product size x = 100 μm, y = 100 μm, z = 20 μm. During the melting of the product support, the laser power is 100 W, when the product itself is melted −150 W, the scanning speed is 380 mm/s. The stainless steel powder 17-4PH, dispersed from 0.5 to 40 μm, made by 3D Systems, was used in the experiments.

The femur bones of rabbits were used. After demineralization of the rabbit femur bones, a lattice endoprosthesis was mounted in. The resulting construct was transplanted under the skin of the back of healthy rabbits. After 45 and 110 days, an X-ray was made and the construction was extracted. The transplanted bone fragments were fixed in 10% formalin and embedded in paraffin according to the standard method. Sections were stained with hematoxylin-eosin. The histology of bone tissue was analyzed [25,26]. All experiments were performed according to bioethical standards and were approved by the local ethical committee of the Kazan Federal University (Protocol meeting No.2 of 5 May 2015). Bioethical standards correspond to the Directive of the European Parliament and of the Council of 22 September 2010 on the protection of animals used for scientific purposes (Directive 2010/63/UE on the protection of animals used for scientific purposes).

## 3. Results

### 3.1. Elementary Cell and Influence Function

**Figure 4:**
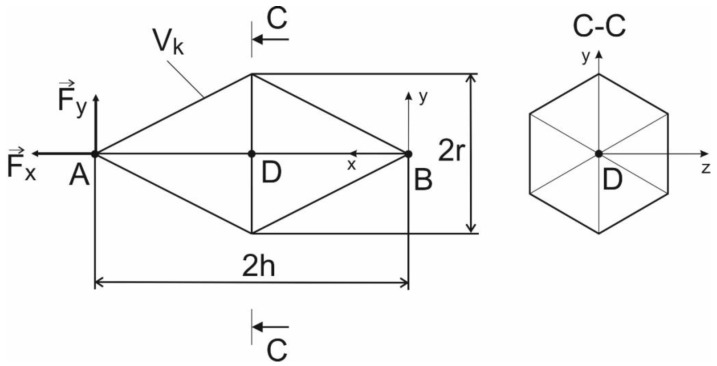
Calculation scheme for a unit cell.

For the convenience of the analysis, a dimensionless cell parameter λ was introduced, determined by the ratio of the cell length to the radius of the circumscribed circle (λ = h/r, Figure 4). The cross-section of the lines forming the cell was assumed to be circular with a diameter of 0.2 mm. In the calculations, a one-dimensional three-node finite element with a quadratic approximation was used. The PS4542A stainless steel 17-4 PH was used for manufacturing. Properties were taken from the official datasheet: Young’s modulus 210 GPa, Poisson’s ratio 0.3, yield point 750 MPa. Further calculations of the cell SSS on compression and bending were carried out for different values of the dimensionless parameter. The parameter varied from 0.25 to 2, in increments of 0.175. To find the influence function multipoint approximation method for solving a mixed integer-continuous optimization, problems were used [27]. To solve the mechanical properties of the lattice cell the method of the representative sample was used [28,29].

The general task of the cell SSS can be formulated as follows:

(6) $$\nabla \tilde{\sigma }=0\;\forall x\in V_{k}$$

(7) $$\tilde{\varepsilon }=\frac{1}{2}\left(\nabla \vec{u}+\nabla {\vec{u}}^{T}\right)\;\forall x\in {\bar{V}}_{k}$$

(8) $$\tilde{\sigma }=\tilde{\tilde{E}}:\tilde{\varepsilon }\;\forall x\in \bar{V}$$

(9) $$\vec{u}=0\;x=0$$

(10) $$\tilde{\sigma }\cdot \vec{n}=\vec{F}\;x=L$$

where *V_k_*—the investigated volume, *σ*—stress tensor, *ε*—strain tensor, *E*—stiffness tensor, *u*—displacement vector, *n*—normal to the area with static boundary conditions.

Point B is rigidly fixed, load F is applied to point A. Two cases of loading were considered: Uniaxial compression and bending. In the calculations for both loading cases, a force of 1 N was used. To estimate the convergence of the finite element mesh, models with a different mesh density were considered. The number of elements on the line varied from 30 to 10 elements. The difference in the results for stress between 30 and 10 elements was less than 1%. The maximum magnitude of the displacement was about 0.2–0.3 mm. The localization of the maximum von Mises stresses in different load cases have not changed, but the magnitude of the maximum stress increased. To estimate the influence of the cell parameter on the maximum von Mises stresses, diagrams were constructed and approximated. It can be concluded that under uniaxial compression, the maximum von Mises stress decreases nonlinearly, and in case of bending, the maximum von Mises stress increases linearly. The influence functions were constructed:

(11) $$y_{u.comp.}=2.3\cdot x^{-1;}\;R^{2}=0.97$$

(12) $$y_{bend.}=30\cdot x-4;\;R^{2}=0.99$$

It can be concluded that the bending force exerts the greatest influence on the strength properties of an elementary cell.

### 3.2. Implant Design

Next, a long bone endoprosthesis was designed. Dimensions and operational loads were selected from the condition of clinical trials of the endoprosthesis on rabbits. Loads were taken equal to F = (−3,1,7) N. Parameters of the implant are given in Table 1. In the calculations, a one-dimensional three-node finite element with a quadratic approximation was used. A general formulation is given in the Materials and Methods chapter.

The algorithm for designing the implant was clarified:

(1) Calculate the SSS for a given state;
(2) Save maximum von Mises stress for every block in the array;
(3) Sort the array in descending order;
(4) Increase the parameter for the first half of the blocks and reduce the parameter for the second half by a given increment value;
(5) Decrease the increment of the parameter;
(6) Check the stop condition otherwise, return to the first step.

In calculations, the increment of the parameter decreased linearly. The stop condition of the iterative process was the permissible number of iterations and the discrepancy of the greatest stresses in the blocks:

(13) $$\left|\underset{i}{\max}\sigma_{i}^{\max}-\underset{i}{\min}\sigma_{i}^{\max}\right|<\varepsilon_{\sigma }$$

**Figure 5:**
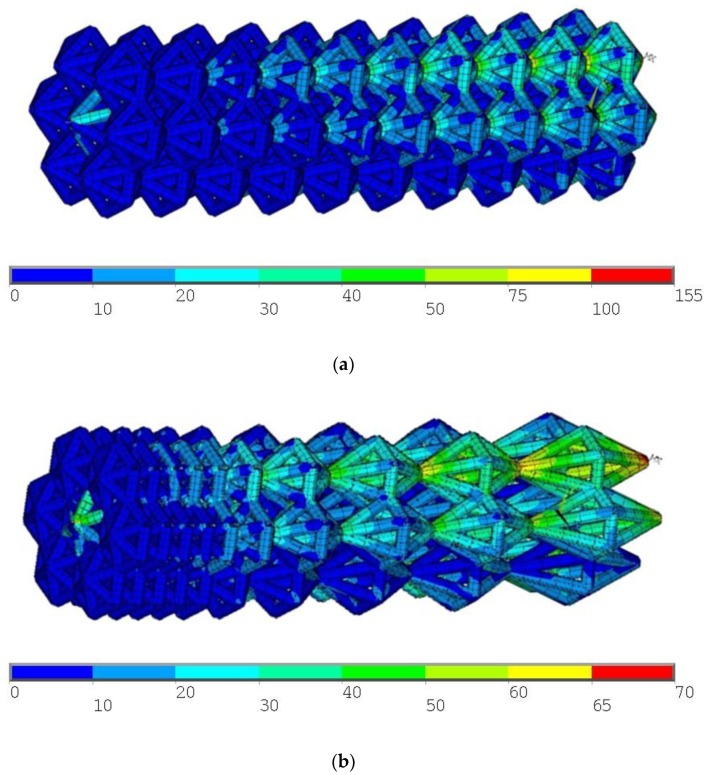
Initial design of the endoprosthesis (**a**) and optimized design of the endoprosthesis (**b**).

The initial increment value was 0.3, the maximum number of iterations—15. In calculation, the symmetry of the implant was used. The distribution of von Mises stresses for the initial design is shown in Figure 5a. The maximum von Mises stress occurs near the seats of the implant and equal to 155 MPa (see Figure 5a).

The implant was optimized by the proposed algorithm. To improve the manufacturing of the design, the results for linear dimensions were standardized according to the recommended numbers Ra40. In this case, the parameter values were: λ = (0.4, 0.4, 0.4, 0.4., 0.5, 0.8, 1.3, 1.7, 1.8, 2.3). The maximum stresses for Mises, in this case, were 71.3 MPa (see Figure 5b). The localization of maximum stresses was near the seats of the implant.

### 3.3. Manufacturing and Clinic Research

The process of selective laser melting depends on various parameters, such as power and scanning speed of the laser, the powder thickness, the orientation of the fabricated structure, the supports location. The correct arrangement of supports is necessary for the effective removal of the released heat during laser melting. During the scan, the laser power is 20% of the total power for supports and 30% for the design itself. The laser scanning speed of the powder surface was 0.4 m/s. The implant design was grown in three different directions: Horizontal placement, vertical (90°), vertical at an angle of 60° to the surface of the base platform, and vertical at an angle of 45°.

In the experiment, two groups were used: Control group (n = 6) and experimental group (n = 6). The weight of the animals were about 2800–3200 g. Under anesthesia, after treating the surgical field with a 70% alcohol solution, a skin incision 3 cm long was made on the back. Then, a lattice endoprosthesis was mounted in decalcified rabbit femur. The wound was sutured in layers. Skin sutures were treated with a 5% alcoholic iodine solution. An aseptic dressing was applied. In the control group, the transplanted bone was without endoprosthesis. In this case, we assume that decalcification provides the best conditions for the migration of cells into the bone. For both groups, histology analysis was made on the 45th and 110th days.

**Figure 6:**
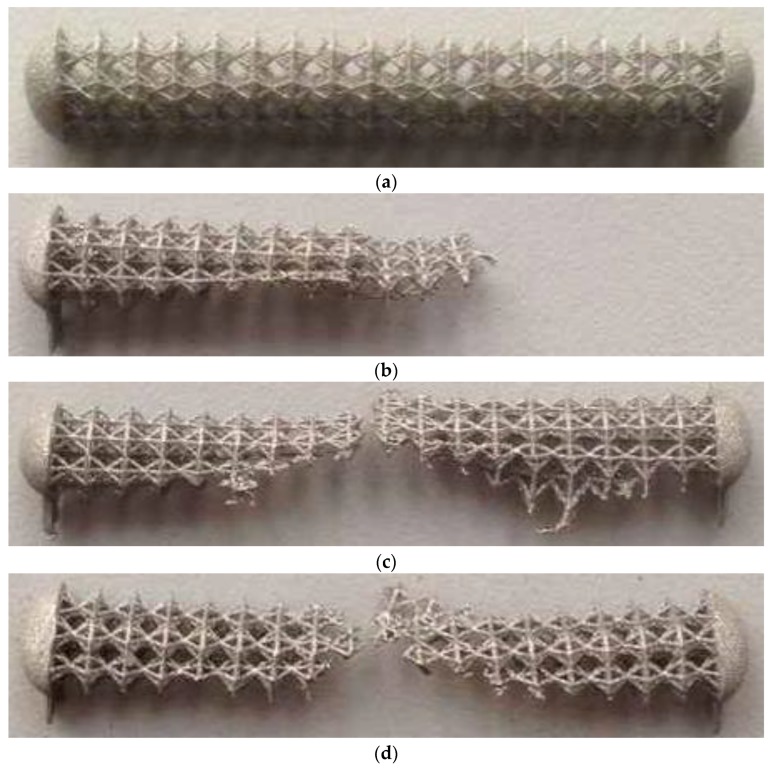
Results of manufacturing: Growth with the angle of 45° to vertical (**a**), growth with vertical orientation (**b**), growth with horizontal orientation (**c**), growth with the angle of 60° to vertical (**d**).

As a result of the experiments, it was found that the most correct arrangement corresponds to a vertical one at an angle of 45° to the cultivated surface (see Figure 6a). When the sample was placed vertically, a negative result was observed, since the sintering of the structure did not occur, or it was not strong and collapsed when the next layer of powder was applied with a metal roller (see Figure 6b). With the horizontal placement of the sample, partial destruction of the lower side of the implant occurred (see Figure 6c). When grown at an angle of 60°, the destruction of the upper part of the implant is observed (see Figure 6d). The observed effect most likely consists of the relative location of the ribs of the designed structure and the appearance of areas in which cross-scanning takes place, leading to the phenomenon of remelting and intense release of thermal energy.

**Figure 7:**
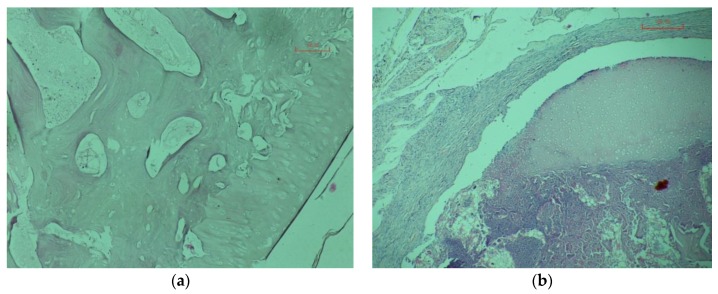
Micrograph of bone X400: The 110 days after transplantation, control animal (**a**); 110 days after transplantation, experienced animal (**b**).

On the 45th day after transplantation in animals of the control and experimental groups in the transplanted bones, the cells are not determined (see Figure 7a).

On the 110th day after transplantation in animals of the control and experimental groups above and to the left is the connective tissue surrounding the graft. The transplanted bone is represented by a site of hyaline cartilage (above) to which from the inside a portion of the resorbable bone matrix is adjacent. A similar picture is observed both in the control and in the experimental group (see Figure 7b).

The results of a morphological study indicate the maintenance of the diffusion of substances and the migration of cells through a worked-out construct.

## 4. Conclusions

The algorithm for designing a long-bone mesh implant is proposed. In the framework of the study, a hexagonal bipyramid was chosen for the elementary cell. The influence function was restored.

The following assumptions were used: Calculation was carried out in an elastic zone, the blocks were loaded uniformly in the radial direction.

The iterative process was realized by the linear reduction of the increment value. The proposed approach is not successful since the increment rapidly decreases to a small value.

The implant was designed for the given parameters. The stress–strain state was calculated for the initial and optimized construction. The comparison of maximum von Mises stress was made for both constructions. The maximum von Mises stress for optimized construction was less than the initial by 53%.

The most correct arrangement in additive manufacturing corresponds to a vertical one at an angle of 45° to the cultivated surface.

The connective tissue surrounding the graft starts to grow after the 110th day.

## Figures and Tables

**Table 1:** Geometric and mechanical characteristics.

Name of Parameter	Value
Length of the implant	40 mm
Width of the implant	6 mm
The radius of the cross-section of the rod	0.2 mm
Material	Steel 17-4 PH
The initial value of the dimensionless parameter	1
Number of blocks	20
Number of cells in the block	7
